# A Revisit of Electromagnetic Wave Scattering by a Metal Isotropic Body in a Lossless Environment with Magnetic Sensor Excitation

**DOI:** 10.3390/s24123807

**Published:** 2024-06-12

**Authors:** Panayiotis Vafeas

**Affiliations:** Department of Chemical Engineering, University of Patras, 26504 Patras, Greece; vafeas@chemeng.upatras.gr; Tel.: +30-2610-996872

**Keywords:** electromagnetic fields, low-frequency scattering, magnetic source, spherical coordinates

## Abstract

This paper investigates the electromagnetic fields being scattered by a metal spherical object in a vacuum environment, providing a numerical implementation of the obtained analytical results. A time-harmonic magnetic dipole source, far enough, emits the incident field at low frequencies, oriented arbitrarily in the three-dimensional space. The aim is to find a detailed solution to the scattering problem at spherical coordinates, which is useful for data inversion. Based on the theory of low frequencies, the Maxwell-type problem is transformed into Laplace’s or Poisson’s interconnected equations, accompanied by the proper boundary conditions on the perfectly conducting sphere and the radiation conditions at infinity, which are solved gradually. Broadly, the static and the first three dynamic terms are sufficient, while the terms of a higher order are negligible, which is confirmed by the field graphical representation.

## 1. Introduction

Electromagnetic waves have been extensively used in medical settings for diagnostic purposes, such as for the detection of cancerous tissues, stroke events or cardiovascular risk, as the behavior of the waves upon meeting their target gives pertinent information for diagnostic and imaging purposes. On the other hand, regarding the Earth’s subsurface electromagnetic probing, especially in deep mineral exploration, the existence of cavities prevent the smooth elaboration of the process and workers must know a priori the location, orientation, size and shape of such anomalies. However, this inversion task cannot be tackled in a robust fashion unless proper analytical models of the electromagnetic field interaction are available in order to bring good insight to the wave field behavior and provide the adequate information for an inversion algorithm.

Consequently, applications related to the response of arbitrary-shaped scatterers in various media, when stimulated by primary sources, stand in the frontline of the current science. Such situations are solved as electromagnetic wave scattering problems, of which there are two types. The first one concerns the forward problem, aiming to determine the scattered field via the corresponding boundary value problems of wave propagation, knowing the physical and the geometrical properties of the scatterer. The second one refers to the inverse problem, where we seek information about the nature of the scatterer, knowing its effect on the wave field. However, of greater interest is the inverse problem [1,2], which becomes particularly difficult if there is no prior knowledge of the corresponding forward problem. Hence, towards this direction, we understand the necessity to obtain efficient models, using Maxwell’s fundamental principles of electromagnetism [3] and the very-well-developed low-frequency scattering theory [4].

Traveling along the history, we indicatively present several references, taken from the already ample literature, which deal with mathematical wave scattering problems, whose solutions show the complexity of the geometrical configuration [5] and the analytical techniques due to the associated functions [6]. Indeed, the initial motivation of this work comes from fundamental applications, such as the detection of inclusions in two-phase composites [7], the Earth’s subsurface electromagnetic probing for mineral exploration [8], the identification of cavities [9], other underground detections for UneXploded Ordinance [10] and buried objects [11], scattering by chiral bodies in chiral or non-chiral surrounding [12] and many other cases related to the retrieval of more complex materials of different shapes and sizes independently of the surrounding environment. This shows the intense necessity of introducing simple, but at the same time, reliable analytical models in the field, even for completely symmetrical shapes. We especially refer to the study of the low-frequency scattering from perfectly conducted complete isotropic bodies (e.g., see [13]) which are embedded in a conductive environment and based on these analytical outcomes. An important work [14] demonstrates the efficiency of the methodology by providing an effective solution of the inverse problem. Within this concept, more complicated geometries for the impenetrable scatterers have been investigated, like two almost-touching spheres [15] or toroidal-shaped bodies [16]. On the other hand, aspects dealing with integral methods are also applicable in suchlike analytical frameworks; for example, an inverse scheme is used to localize a smooth surface of a three-dimensional perfectly conducting object using a boundary integral formulation in [17]. Otherwise, similar models with respect to the low-frequency scattering by metallic objects of a spherical geometry [18,19,20], but also from different geometries [21,22,23,24], which are surrounded by a lossless medium, are very useful since they find direct important applications.

In this project, we intend to provide a better insight into the different structures of a solid metal scatterer in a lossless surrounding, when this body is illuminated by a magnetic dipole source that operates at low frequencies. We revisit the interesting case of the geometrically complete isotropic scatterer, as a potential application to real-life problems and non-invasive techniques. Therein, we solve analytically the forward electromagnetic scattering problem in the low-frequency regime with the appropriately chosen conditions on the non-penetrable sphere and at infinity. Hence, we arrive to a sequence of boundary value problems, involving Laplace’s and Poisson’s interconnected equations, whose solution provides us with the scattered fields in terms of spherical harmonic eigenfunctions [5,6]. Towards the direction of verifying the analytical formulae, the complicated analysis is supplemented by the proper numerical implementation, which is provided via a graph illustration of the scattered magnetic field, which is the measureable field in such physical applications.

## 2. Theoretical Development

**Figure 1:**
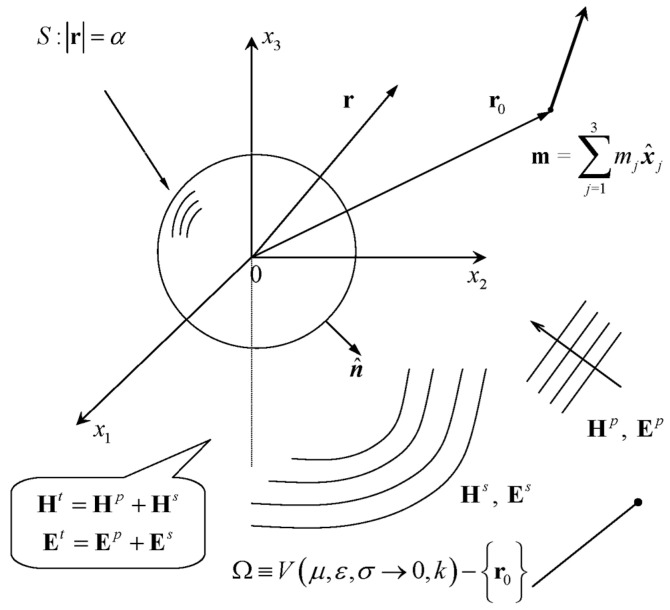
Representation of the scattering problem.

The geometrical configuration of the scattering problem under consideration is shown in Figure 1. In view of the Cartesian basis $\left({\hat{x}}_{1},{\hat{x}}_{2},{\hat{x}}_{3}\right)$, we define the electric field $E^{x}$ and the magnetic field $H^{x}$, where x=p,s,t denote the primary (also referred as incident) p, the scattered s and the total t electromagnetic fields, accounting for the fact that $E^{t}\left(r\right)=E^{p}\left(r\right)+E^{s}\left(r\right)$ and $H^{t}\left(r\right)=H^{p}\left(r\right)+H^{s}\left(r\right)$. Here, $r=x_{1}{\hat{x}}_{1}+x_{2}{\hat{x}}_{2}+x_{3}{\hat{x}}_{3}$ is the spatial position vector, while $r_{0}$ stands for the position of the magnetic dipole m, which radiates at a low circular frequency ω. The surface S of the impenetrable ($\sigma_{b}\to +\infty$) spherical scatterer of radius α is characterized by the outward unit normal vector $\hat{n}$, while the properties of the surrounding medium are the dielectric permittivity ε, the magnetic permeability μ and the electric conductivity σ→0 (lossless medium), being connected via the wave number $k=\omega \sqrt{\varepsilon \mu }$ in terms of the operating frequency. The area of electromagnetic scattering is confined by Ω, assumed to be

(1) $$\Omega \equiv V\left({\mathbb{R}}^{3}\right)-\left\{r_{0}\right\}$$

excluding the singular point of the source position, whose arbitrary orientation is determined by the relation

(2) $$m=\sum_{j=1}^{3}m_{j}{\hat{x}}_{j}$$

The involved electromagnetic fields satisfy Maxwell’s equations in view of the ∇ and Δ operators [5],

(3) $$\nabla \times E^{x}\left(r\right)=i\omega \mu \;H^{x}\left(r\right)\;\mathrm{and}\;\nabla \times H^{x}\left(r\right)=-i\omega \varepsilon \;E^{x}\left(r\right)\;\mathrm{with}\;\;\nabla \cdot H^{x}\left(r\right)=\nabla \cdot E^{x}\left(r\right)=0$$

for every r∈Ω and x=p,s,t, providing the Helmholtz equations

(4) $$\left(\Delta +k^{2}\right)E^{x}\left(r\right)=\left(\Delta +k^{2}\right)H^{x}\left(r\right)=0,\;r\in \Omega$$

with boundary conditions on the spherical surface

(5) $$\hat{n}\cdot H^{t}\left(r\right)=0\;\mathrm{and}\;\hat{n}\times E^{t}\left(r\right)=0,\;r\in S$$

for x=p,s,t, canceling the normal component of the total magnetic field and the tangential components of the total electric field. The radiation Silver–Müller conditions for the scattered fields are given by

(6) $$\underset{\left|r\right|\to +\infty }{\lim}\left[r\times \nabla \times \left(\begin{array}{l} H^{s}\left(r\right)\\ E^{s}\left(r\right) \end{array}\right)+ik\left|r\right|\left(\begin{array}{l} H^{s}\left(r\right)\\ E^{s}\left(r\right) \end{array}\right)\right]=0,\;r\in \Omega ,$$

securing the proper behavior of the fields at infinity.

Within the framework of the low-frequency theory, we expand all the fields in terms of the positive integral powers of $\left(ik\right)$, wherein i is the imaginary unit. Therefore,

(7) $$H^{x}\left(r\right)=\sum_{n=0}^{+\infty }H_{n}^{x}\left(r\right)\frac{{\left(ik\right)}^{n}}{n!},\;r\in \Omega \;\mathrm{for}\;x=p,s,t$$

and

(8) $$E^{x}\left(r\right)=\sum_{n=0}^{+\infty }E_{n}^{x}\left(r\right)\frac{{\left(ik\right)}^{n}}{n!},\;r\in \Omega \;\mathrm{for}\;x=p,s,t.$$

As ω is considered very low, the term $\left(ik\right)$ becomes low as well, thus when n∈ℕ increases, then ${\left(ik\right)}^{n}$ decreases rapidly. Therefore, without loss of generality, we restrict ourselves to orders for n=0,1,2,3, since the terms of higher orders (n≥4) can be omitted. The low-frequency expansions of the incident fields are known (see also Appendix A for technical details) and they imply that the surviving (non-zero) electric and magnetic scattered fields are $H_{0}^{s},\;H_{2}^{s},\;H_{3}^{s},\;E_{1}^{s},\;E_{3}^{s}$ (due to the fact that $H_{1}^{p}=E_{0}^{p}=E_{2}^{p}=0$, it holds $H_{1}^{s}=E_{0}^{s}=E_{2}^{s}=0$), which are the requested scattered electromagnetic fields, being calculated on the basis of the analysis that follows.

Substituting the expansions (7) and (8) into the fundamental Equation (3) and the conditions (5) and (6), we arrive to a complicated sequence of boundary value problems for the scattered fields. Actually, the first task is to reduce the Maxwell’s relations (3) for x=s to the corresponding low-frequency counterparts, i.e.,

(9) $$\nabla \times E_{n}^{s}\left(r\right)=n\sqrt{\frac{\mu }{\varepsilon }}H_{n-1}^{s}\left(r\right),\;n\geq 0$$

and

(10) $$\nabla \times H_{n}^{s}\left(r\right)=-n\sqrt{\frac{\varepsilon }{\mu }}E_{n-1}^{s}\left(r\right),\;n\geq 0,$$

While, due to (3), it holds $\nabla \cdot E_{n}^{s}=\nabla \cdot H_{n}^{s}=0$ for n≥0. Thereafter, we are led to the interconnected partial differential equations for the surviving electromagnetic fields for each order of interest, i.e., n=0,1,2,3, using classical vector identities. To this end, we initially apply the ∇× on both sides of (10) for n=0, so we obtain

(11) $$\begin{array}{cc} \nabla \times \nabla \times H_{0}^{s}\left(r\right)=0\; & \Rightarrow \;\nabla \nabla \cdot H_{0}^{s}\left(r\right)-\Delta H_{0}^{s}\left(r\right)=0\\ & \Rightarrow \;\Delta H_{0}^{s}\left(r\right)=0\Rightarrow \;H_{0}^{s}\left(r\right)=\nabla \Phi_{0}^{s}\left(r\right), \end{array}$$

since $\nabla \cdot H_{0}^{s}=0$ and $\nabla \times H_{0}^{s}=0$ (see (10) for instance), where $\Phi_{0}^{s}$ is an arbitrary scalar harmonic function. In the sequel, we work similarly on (10) for n=2 and we also use (9) for n=1, as well as the previous outcome (11), in order to calculate

(12) $$\begin{array}{cc} \nabla \times \nabla \times H_{2}^{s}\left(r\right)=-2\sqrt{\frac{\varepsilon }{\mu }}\nabla \times E_{1}^{s}\left(r\right)\; & \Rightarrow \;\nabla \nabla \cdot H_{2}^{s}\left(r\right)-\Delta H_{2}^{s}\left(r\right)=-2\sqrt{\frac{\varepsilon }{\mu }}\sqrt{\frac{\mu }{\varepsilon }}H_{0}^{s}\left(r\right)\\ & \Rightarrow \;\Delta H_{2}^{s}\left(r\right)=2H_{0}^{s}\left(r\right)\;\Rightarrow \;\Delta H_{2}^{s}\left(r\right)=2\nabla \Phi_{0}^{s}\left(r\right)\\ & \Rightarrow \;H_{2}^{s}\left(r\right)=X_{2}^{s}\left(r\right)+r\Phi_{0}^{s}\left(r\right), \end{array}$$

since $\nabla \cdot H_{2}^{s}=0$, where $X_{2}^{s}$ is an arbitrary vector harmonic function. The last result into (12) is the summation of a general harmonic solution and of a particular solution $r\Phi_{0}^{s}$, which holds because it is $\Delta \left(r\Phi_{0}^{s}\right)=\Phi_{0}^{s}\Delta r+r\Delta \Phi_{0}^{s}+2\left(\nabla ⊗r\right)\cdot \nabla \Phi_{0}^{s}=2\tilde{I}\cdot \nabla \Phi_{0}^{s}=2\nabla \Phi_{0}^{s}$, noting that $\nabla ⊗r=\tilde{I}$ and Δr=0, $\Delta \Phi_{0}^{s}=0$; consequently, $\Delta H_{2}^{s}=2\nabla \Phi_{0}^{s}$, where the symbol “⊗” stands for the classical dyadic product, while $\tilde{I}$ denotes the unit dyadic. Next, once again, we apply the ∇× on both sides of (10) for n=3 and we combine it with (9) for n=2 to conclude

(13) $$\begin{array}{cc} \nabla \times \nabla \times H_{3}^{s}\left(r\right)=-3\sqrt{\frac{\varepsilon }{\mu }}\nabla \times E_{2}^{s}\left(r\right)\; & \Rightarrow \;\nabla \nabla \cdot H_{3}^{s}\left(r\right)-\Delta H_{3}^{s}\left(r\right)=-6\sqrt{\frac{\varepsilon }{\mu }}\sqrt{\frac{\mu }{\varepsilon }}H_{1}^{s}\left(r\right)\\ & \Rightarrow \;\Delta H_{3}^{s}\left(r\right)=0\;\Rightarrow \;H_{3}^{s}\left(r\right)=\nabla \Phi_{3}^{s}\left(r\right), \end{array}$$

since $\nabla \cdot H_{3}^{s}=0$ and $\nabla \times H_{3}^{s}=0$ (see (10), for example, in which $E_{2}^{s}=0$ in the absence of an incident field), where $\Phi_{3}^{s}$ is an arbitrary scalar harmonic function, while we have used the fact that $H_{1}^{s}=0$, due to the lack of the corresponding incident field. Proceeding the remaining electric scattered fields, it is trivial to recover $E_{1}^{s}$ from (10) for n=2, hence

(14) $$E_{1}^{s}\left(r\right)=-\frac{1}{2}\sqrt{\frac{\mu }{\varepsilon }}\nabla \times H_{2}^{s}\left(r\right),$$

wherein $H_{2}^{s}$ is a known field from (12). Finally, the last surviving electric low-frequency component is found by applying the ∇× on both sides of (9) for n=3 and, at the same time, using (10) for n=2, so

(15) $$\begin{array}{cc} \nabla \times \nabla \times E_{3}^{s}\left(r\right)=3\sqrt{\frac{\mu }{\varepsilon }}\nabla \times H_{2}^{s}\left(r\right)\; & \Rightarrow \;\nabla \nabla \cdot E_{3}^{s}\left(r\right)-\Delta E_{3}^{s}\left(r\right)=-6\sqrt{\frac{\mu }{\varepsilon }}\sqrt{\frac{\varepsilon }{\mu }}E_{1}^{s}\left(r\right)\\ & \Rightarrow \;\Delta E_{3}^{s}\left(r\right)=6E_{1}^{s}\left(r\right)\\ & \Rightarrow \;E_{3}^{s}\left(r\right)=X_{3}^{s}\left(r\right)+6\left[-\frac{1}{4\pi }\underset{\Omega }{∭}\frac{E_{1}^{s}\left(r^{\prime }\right)}{\left|r-r^{\prime }\right|}d\Omega^{\prime }\right], \end{array}$$

since $\nabla \cdot E_{3}^{s}=0$, where $X_{3}^{s}$ is an arbitrary vector harmonic function, while $E_{1}^{s}$ is known from (14). The last result into (15) is the summation of a general harmonic part and of a particular part, which is written in terms of the fundamental solution of the Laplace’s operator $\Delta \left[-1/4\pi \left|r-r^{\prime }\right|\right]=\delta \left(r-r^{\prime }\right)$, in view of the Dirac measure δ. Eventually, all the active electromagnetic fields will have been calculated within (11)–(15) by means of the easy-to-handle harmonic scalar $\Phi_{0}^{s}$, $\Phi_{3}^{s}$ and vector $X_{2}^{s}$, $X_{3}^{s}$ functions, those being

(16) $$\Delta \Phi_{0}^{s}\left(r\right)=0,\;\Delta X_{2}^{s}\left(r\right)=0,\;\Delta \Phi_{3}^{s}\left(r\right)=0,\;\Delta X_{3}^{s}\left(r\right)=0$$

for any r∈Ω, while the second term on the right-hand side of (15) is an immediate consequence of the fundamental solution of Laplace’s equation [6]. On the other hand, the surface boundary conditions (5) become

(17) $$\hat{n}\cdot H_{n}^{t}\left(r\right)\equiv \hat{n}\cdot \left[H_{n}^{p}\left(r;r_{0}\right)+H_{n}^{s}\left(r\right)\right]=0,\;n=0,2,3,$$

and

(18) $$\hat{n}\times E_{n}^{t}\left(r\right)\equiv \hat{n}\times \left[E_{n}^{p}\left(r;r_{0}\right)+E_{n}^{s}\left(r\right)\right]=0,\;n=1,3,$$

whilst the infinity conditions yield is

(19) $$\underset{\left|r\right|\to +\infty }{\lim}\left[r\times \nabla \times \left(\begin{array}{l} H_{n}^{s}\left(r\right)\\ E_{n}^{s}\left(r\right) \end{array}\right)+n\left|r\right|\left(\begin{array}{l} H_{n-1}^{s}\left(r\right)\\ E_{n-1}^{s}\left(r\right) \end{array}\right)\right]=0,\;r\in \Omega .$$

Our goal is to solve the incorporated boundary value problems (11)–(19) by introducing the best-fitted spherical geometry [5] $r=r\zeta {\hat{x}}_{1}+r\sqrt{1-\zeta^{2}}\cos\varphi {\hat{x}}_{2}+r\sqrt{1-\zeta^{2}}\sin\varphi {\hat{x}}_{3}$, where $r\in \left[0,+\infty \right)$, $\zeta \equiv \cos\theta \in \left[-1,1\right]$ and $\varphi \in \left[0,2\pi \right]$ with the outward unit normal vector $\hat{n}\equiv \hat{r}=r/r$.

## 3. Spherical Scattered Fields

In order to proceed to the solution, we are obliged to present the basic mathematical tools, which are used in this project [6]. Bearing this in mind, we initially give the expansion of any harmonic function $u\left(r\right)$ (either scalar or vector) that belongs to the kernel space of the Laplace’s operator ($\Delta u\left(r\right)=0$), that is

(20) $$u\left(r\right)=\sum_{l=0}^{+\infty }\sum_{m=0}^{l}\left[A_{l,in}^{m/q}u_{l,in}^{m/q}\left(r\right)+A_{l,ex}^{m/q}u_{l,ex}^{m/q}\left(r\right)\right]$$

for $r\in \left\{r\in \left[0,+\infty \right),\;\zeta \in \left[-1,1\right],\;\varphi \in \left[0,2\pi \right)\right\}$. Expansion (20) is a linear combination (in which $A_{l,in}^{m/q}$, $A_{l,ex}^{m/q}$ are arbitrary constant coefficients) with respect to the functions

(21) $$u_{l,in}^{m/q}\left(r\right)=r^{l}Y_{l}^{m/q}\left(\zeta ,\varphi \right),\;r\in \left\{r\in \left[0,+\infty \right),\;\zeta \in \left[-1,1\right],\;\varphi \in \left[0,2\pi \right)\right\}$$

and

(22) $$u_{l,ex}^{m/q}\left(r\right)=r^{-\left(l+1\right)}Y_{l}^{m/q}\left(\zeta ,\varphi \right),\;r\in \left\{r\in \left[0,+\infty \right),\;\zeta \in \left[-1,1\right],\;\varphi \in \left[0,2\pi \right)\right\},$$

which define the respective interior and exterior spherical harmonic eigenfunctions of the degree l≥0 and order m=0,1,2,…,l (note that q=e,o), written in terms of the surface spherical harmonics

(23) $$Y_{l}^{m/q}\left(\zeta ,\varphi \right)=P_{l}^{m}\left(\zeta \right)f_{m}^{q}\left(\varphi \right),$$

in view of the associated Legendre functions of the first kind $P_{l}^{m}\left(\zeta \right)$, $\zeta \in \left[-1,1\right]$, where

(24) $$f_{m}^{q}\left(\varphi \right)=\left\{\begin{array}{l} \cos m\varphi ,\;q=e\\ \sin m\varphi ,\;q=o \end{array}\right.,\;\varphi \in \left[0,2\pi \right)$$

stand for the even (q=e) and the odd (q=o) trigonometric functions. The surface spherical harmonic functions $Y_{l}^{m/q}\left(\zeta ,\varphi \right)$ are orthogonal with respect to the surface integral

(25) $$\int_{0}^{2\pi }\int_{-1}^{+1}Y_{l}^{m/q}\left(\zeta ,\varphi \right)Y_{l^{\prime }}^{m^{\prime }/q^{\prime }}\left(\zeta ,\varphi \right)d\zeta d\varphi =\frac{1}{\varepsilon_{m}}\frac{4\pi }{2l+1}\frac{\left(l+m\right)!}{\left(l-m\right)!}\delta_{ll^{\prime }}\delta_{mm^{\prime }}\delta_{qq^{\prime }}$$

for any l≥0, m=0,1,2,…,l and q=e,o. In addition, the following expansion

(26) $$\frac{1}{R}=\frac{1}{\left|r-r_{0}\right|}=\sum_{l=0}^{+\infty }\sum_{m=0}^{l}\sum_{q=e,o}\rho_{l,ex}^{m/q}\left(r_{0}\right)u_{l,in}^{m/q}\left(r\right),\;r<r_{0}$$

with

(27) $$\rho_{l,ex}^{m/q}\left(r_{0}\right)=\frac{\left(l-m\right)!}{\left(l+m\right)!}\varepsilon_{m}u_{l,ex}^{m/q}\left(r_{0}\right)=\frac{\left(l-m\right)!}{\left(l+m\right)!}\varepsilon_{m}r_{0}^{-\left(l+1\right)}Y_{l}^{m/q}\left(\zeta_{0},\varphi_{0}\right)$$

for l≥0, m=0,1,2,…,l and q=e,o is useful to our calculations in the domains, wherein the singular point is far away from the scattering region ($r<r_{0}$), as in our case.

Turning, now, to our specific problem, we are reminded of the fact that the region of electromagnetic activity Ω is confined by the set

(28) $$\Omega =\left\{\left(r,\zeta ,\varphi \right):r\in \left[\alpha ,+\infty \right),\;\zeta \in \left[-1,1\right],\;\varphi \in \left[0,2\pi \right)\right\}-\left\{r_{0}\right\}$$

restricting ourselves to an exterior-type problem. Hence, the general expansion (20) is reduced accordingly to attain the proper behavior at infinity, by setting $A_{l,in}^{m/q}=0$ herein and in every similar expansion in the forthcoming analysis. In what follows, we readily define as $r_{s}=\left(r_{s},\zeta ,\varphi \right)\equiv \left(\alpha ,\zeta ,\varphi \right)$ and as $r_{0}=\left(r_{0},\zeta_{0},\varphi_{0}\right)$ the position vectors pointing on the surface of the spherical body and at the position of the dipole source, while we omit presenting the full analysis (for more details we refer to [18]), since the calculations, based on (20)–(27) are cumbersome and out of the spirit of this research.

We begin with the evaluation of Rayleigh field $H_{0}^{s}$, whose solution is given through the exterior scalar harmonic potential

(29) $$\Phi_{0}^{s}\left(r\right)=\sum_{l=0}^{+\infty }\sum_{m=0}^{l}\sum_{q=e,o}a_{l,ex}^{m/q}r^{-\left(l+1\right)}Y_{l}^{m/q}\left(\zeta ,\varphi \right)\;\mathrm{for}\;r\in \Omega ,$$

via (11), wherein $a_{l,ex}^{m/q}$ for l≥0, m=0,1,2,…,l and q=e,o denote the unknown constant coefficients that must be determined from the boundary condition (17) for n=0 on the sphere r=α. Towards this direction, we initially use (11) with (29) to evaluate

(30) $$\hat{r}\cdot H_{0}^{s}\left(\alpha ,\zeta ,\varphi \right)=-\sum_{l=0}^{\infty }\sum_{m=0}^{l}\sum_{q=e,o}a_{l,ex}^{m/q}\left(l+1\right)\alpha^{-\left(l+2\right)}Y_{l}^{m/q}\left(\zeta ,\varphi \right)$$

for $\zeta \in \left[-1,1\right]$, $\varphi \in \left[0,2\pi \right)$, while, in the sequel, we utilize the symmetric form of the incident magnetic field of zero order (see Appendix A) and expansion (26) with (27) to obtain

(31) $$\begin{array}{cc} \hat{r}\cdot H_{0}^{p}\left(\alpha ,\zeta ,\varphi \right) & ={\left\{\frac{\partial }{\partial r}\left[\nabla \frac{1}{\left|r-r_{0}\right|}\right]\cdot \frac{m}{4\pi }\right\}}_{r=\alpha }=-{\left\{\frac{\partial }{\partial r}\left[\nabla_{r_{0}}\frac{1}{\left|r-r_{0}\right|}\right]\cdot \frac{m}{4\pi }\right\}}_{r=\alpha }\\ & =-\sum_{l=0}^{+\infty }\sum_{m=0}^{l}\sum_{q=e,o}\left(\frac{m}{4\pi }\cdot \nabla_{r_{0}}\rho_{l,ex}^{m/q}\left(r_{0}\right)\right)l\alpha^{l-1}Y_{l}^{m/q}\left(\zeta ,\varphi \right) \end{array}$$

for $\zeta \in \left[-1,1\right]$ and $\varphi \in \left[0,2\pi \right)$. Next, we combine (30) and (31) in view of (17) for n=0 and with $\hat{n}\equiv \hat{r}$. Consequently, we are led to

(32) $$\sum_{l=0}^{+\infty }\sum_{m=0}^{l}\sum_{q=e,o}\left[\left(\frac{m}{4\pi }\cdot \nabla_{r_{0}}\rho_{l,ex}^{m/q}\left(r_{0}\right)\right)l\alpha^{l-1}+a_{l,ex}^{m/q}\left(l+1\right)\alpha^{-\left(l+2\right)}\right]Y_{l}^{m/q}\left(\zeta ,\varphi \right)=0$$

for every $\zeta \in \left[-1,1\right]$ and $\varphi \in \left[0,2\pi \right)$, from which orthogonality arguments with respect to (25) imply

(33) $$a_{l,ex}^{m/q}=-\frac{l\alpha^{2l+1}}{l+1}\left(\frac{m}{4\pi }\cdot \nabla_{r_{0}}\rho_{l,ex}^{m/q}\left(r_{0}\right)\right)\;\mathrm{for}\;l\geq 0,\;m=0,1,2,\ldots ,l\;\mathrm{and}\;q=e,o.$$

Substituting the constant coefficients (33) into (29), we recover the relative potential field as

(34) $$\Phi_{0}^{s}\left(r\right)=-\sum_{l=0}^{+\infty }\sum_{m=0}^{l}\sum_{q=e,o}\left[\left(\frac{m}{4\pi }\cdot \nabla_{r_{0}}\rho_{l,ex}^{m/q}\left(r_{0}\right)\right)\frac{l\alpha^{2l+1}}{l+1}r^{-\left(l+1\right)}Y_{l}^{m/q}\left(\zeta ,\varphi \right)\right],$$

Consequently, from relation (11) we obtain

(35) $$\begin{array}{cc} H_{0}^{s}\left(r\right) & =-\sum_{l=0}^{+\infty }\sum_{m=0}^{l}\sum_{q=e,o}\left\{\frac{l\alpha^{2l+1}}{l+1}\frac{\left(l-m\right)!}{\left(l+m\right)!}\varepsilon_{m}\left[\frac{m}{4\pi }\cdot \nabla_{r_{0}}u_{l,ex}^{m/q}\left(r_{0}\right)\right]\nabla u_{l,ex}^{m/q}\left(r\right)\right\}\\ & =-\sum_{l=0}^{+\infty }\sum_{m=0}^{l}\sum_{q=e,o}\left\{\frac{l\alpha^{2l+1}}{l+1}\left[\frac{m}{4\pi }\cdot \nabla_{r_{0}}\rho_{l,ex}^{m/q}\left(r_{0}\right)\right]\nabla \left(r^{-\left(l+1\right)}Y_{l}^{m/q}\left(\zeta ,\varphi \right)\right)\right\} \end{array}$$

for every r∈Ω. Next, we proceed to the $H_{2}^{s}\left(r\right)$, wherein we need the vector harmonic function

(36) $$X_{2}^{s}\left(r\right)=\sum_{l=0}^{\infty }\sum_{m=0}^{l}\sum_{q=e,o}b_{l,ex}^{m/q}r^{-\left(l+1\right)}Y_{l}^{m/q}\left(\zeta ,\varphi \right)$$

and the expansion (26) with (27) in order to come up (via relationship (12)) with the final expansion

(37) $$\begin{array}{cc} H_{2}^{s}\left(r\right) & =\sum_{l=0}^{\infty }\sum_{m=0}^{l}\sum_{q=e,o}\left[b_{l,ex}^{m/q}-\frac{\left(l-m\right)!}{\left(l+m\right)!}\varepsilon_{m}r\left(\frac{m}{4\pi }\cdot \nabla_{r_{0}}u_{l,ex}^{m/q}\left(r_{0}\right)\right)\frac{l\alpha^{2l+1}}{l+1}\right]u_{l,ex}^{m/q}\left(r\right)\\ & =\sum_{l=0}^{\infty }\sum_{m=0}^{l}\sum_{q=e,o}\left[b_{l,ex}^{m/q}-r\left(\frac{m}{4\pi }\cdot \nabla_{r_{0}}\rho_{l,ex}^{m/q}\left(r_{0}\right)\right)\frac{l\alpha^{2l+1}}{l+1}\right]r^{-\left(l+1\right)}Y_{l}^{m/q}\left(\zeta ,\varphi \right) \end{array}$$

for every r∈Ω. The constants $b_{l,ex}^{m/q}=b_{l,1}^{m/q}{\hat{x}}_{1}+b_{l,2}^{m/q}{\hat{x}}_{2}+b_{l,3}^{m/q}{\hat{x}}_{3}$ for any value of l≥0, m=0,1,2,…,l and q=e,o satisfy the three independent relationships

(38) $$\sum_{l=0}^{+\infty }\sum_{m=0}^{l}\sum_{q=e,o}\left[\sum_{j=1}^{3}f_{l,j}^{m/q,\kappa }\left(r_{s},\zeta ,\varphi \right)b_{l,j}^{m/q}-g_{l}^{m/q,\kappa }\left(r_{s},\zeta ,\varphi \;;r_{0}\right)\right]=0,\;\kappa =1,2,3,$$

in which the surface functions $f_{l,j}^{m/q,\kappa }\left(r_{s},\zeta ,\varphi \right)$ and $g_{l}^{m/q,\kappa }\left(r_{s},\zeta ,\varphi \;;r_{0}\right)$ for l≥0, m=0,1,2,…,l and q=e,o have complicated forms in view of $P_{l}^{m}\left(\zeta \right)$ and $f_{m}^{q}\left(\varphi \right)$, where, for reasons of completeness and independence in reading, we provide them herein. Thus, for κ=1, we obtain

(39) $$f_{l,1}^{m/q,1}\left(\zeta ,\varphi \right)=\alpha^{-\left(l+1\right)}\zeta Y_{l}^{m/q}\left(\zeta ,\varphi \right),$$

(40) $$f_{l,2}^{m/q,1}\left(\zeta ,\varphi \right)=\alpha^{-\left(l+1\right)}\sqrt{1-\zeta^{2}}\cos\varphi Y_{l}^{m/q}\left(\zeta ,\varphi \right),$$

(41) $$f_{l,3}^{m/q,1}\left(\zeta ,\varphi \right)=\alpha^{-\left(l+1\right)}\sqrt{1-\zeta^{2}}\sin\varphi Y_{l}^{m/q}\left(\zeta ,\varphi \right)$$

and

(42) $$g_{l}^{m/q,1}\left(\zeta ,\varphi \;;r_{0}\right)=\left\{\rho_{l,ex}^{m/q}\left(r_{0}\right)\frac{m}{4\pi }\cdot \left[-\frac{l}{\alpha }\left(r\left(\alpha ,\zeta ,\varphi \right)-r_{0}\right)+\hat{r}\right]+\frac{l\alpha }{l+1}M_{l,ex}^{m/q}\left(r_{0}\right)\right\}\alpha^{l}Y_{l}^{m/q}\left(\zeta ,\varphi \right),$$

For κ=2, we receive

(43) $$f_{l,1}^{m/q,2}\left(\zeta ,\varphi \right)=\alpha^{-\left(l+2\right)}\left[-\left(l+1\right)\sqrt{1-\zeta^{2}}P_{l}^{m}\left(\zeta \right)-\zeta \sqrt{1-\zeta^{2}}P_{l}^{m^{\prime }}\left(\zeta \right)\right]f_{m}^{q}\left(\varphi \right),$$

(44) $$f_{l,2}^{m/q,2}\left(\zeta ,\varphi \right)=\alpha^{-\left(l+2\right)}\left[\left(l+1\right)\zeta P_{l}^{m}\left(\zeta \right)-\left(1-\zeta^{2}\right)P_{l}^{m^{\prime }}\left(\zeta \right)\right]\cos\varphi f_{m}^{q}\left(\varphi \right),$$

(45) $$f_{l,3}^{m/q,2}\left(\zeta ,\varphi \right)=\alpha^{-\left(l+2\right)}\left[\left(l+1\right)\zeta P_{l}^{m}\left(\zeta \right)-\left(1-\zeta^{2}\right)P_{l}^{m^{\prime }}\left(\zeta \right)\right]\sin\varphi f_{m}^{q}\left(\varphi \right)$$

and

(46) $$g_{l}^{m/q,2}\left(\zeta ,\varphi \;;r_{0}\right)=-\frac{l\alpha^{l}}{l+1}M_{l,ex}^{m/q}\left(r_{0}\right)\sqrt{1-\zeta^{2}}P_{l}^{m^{\prime }}\left(\zeta \right)f_{m}^{q}\left(\varphi \right)+2\left(\hat{r}\times M_{l,ex}^{m/q}\left(r_{0}\right)\cdot \hat{\zeta }\right)\alpha^{l}Y_{l}^{m/q}\left(\zeta ,\varphi \right),$$

Meanwhile, for κ=3, we have

(47) $$f_{l,1}^{m/q,3}\left(\zeta ,\varphi \right)=\alpha^{-\left(l+2\right)}\frac{\zeta }{\sqrt{1-\zeta^{2}}}f_{m}^{q^{\prime }}\left(\varphi \right)P_{l}^{m}\left(\zeta \right),$$

(48) $$f_{l,2}^{m/q,3}\left(\zeta ,\varphi \right)=\alpha^{-\left(l+2\right)}\left[-\left(l+1\right)\sin\varphi f_{m}^{q}\left(\varphi \right)+\cos\varphi f_{m}^{q^{\prime }}\left(\varphi \right)\right]P_{l}^{m}\left(\zeta \right),$$

(49) $$f_{l,3}^{m/q,3}\left(\zeta ,\varphi \right)=\alpha^{-\left(l+2\right)}\left[\left(l+1\right)\cos\varphi f_{m}^{q}\left(\varphi \right)+\sin\varphi f_{m}^{q^{\prime }}\left(\varphi \right)\right]P_{l}^{m}\left(\zeta \right)$$

and

(50) $$g_{l}^{m/q,3}\left(\zeta ,\varphi \;;r_{0}\right)=M_{l,ex}^{m/q}\left(r_{0}\right)\frac{l\alpha^{l}}{l+1}\frac{P_{l}^{m}\left(\zeta \right)}{\sqrt{1-\zeta^{2}}}f_{m}^{q^{\prime }}\left(\varphi \right)+2\left(\hat{r}\times M_{l,ex}^{m/q}\left(r_{0}\right)\cdot \hat{\varphi }\right)\alpha^{l}Y_{l}^{m/q}\left(\zeta ,\varphi \right),$$

which are valid for any $\zeta \in \left[-1,1\right]$ and $\varphi \in \left[0,2\pi \right)$. The convenient functions of the dipole’s position that appear within (42), (46) and (50) imply

(51) $$M_{l,ex}^{m/q}\left(r_{0}\right)=\frac{m}{4\pi }\cdot \nabla_{r_{0}}\rho_{l,ex}^{m/q}\left(r_{0}\right)\;\mathrm{and}\;M_{l,ex}^{m/q}\left(r_{0}\right)=\frac{m}{4\pi }\times \nabla_{r_{0}}\rho_{l,ex}^{m/q}\left(r_{0}\right)$$

for l≥0, m=0,1,2,…,l and q=e,o, while all derivatives denoted by the prime are with respect to the argument. Thereafter, we expand the functions (39)–(50) in terms of surface spherical harmonics. Since they belong to the subspace that they produce, we substitute the result into the relations (38) and the outcome is handled with the aid of orthogonality (25) so as to recover the components $b_{l,j}^{m/q}$ (j=1,2,3) of the constant coefficient $b_{l,ex}^{m/q}$ for l≥0, m=0,1,2,…,l and q=e,o. In the sequel, we move to the presentation of the simple solution of $H_{3}^{s}$, which provides us with

(52) $$\Phi_{3}^{s}\left(r\right)=c_{1,ex}^{0/e}r^{-2}Y_{1}^{0/e}\left(\zeta ,\varphi \right)+c_{1,ex}^{1/e}r^{-2}Y_{1}^{1/e}\left(\zeta ,\varphi \right)+c_{1,ex}^{1/o}r^{-2}Y_{1}^{1/o}\left(\zeta ,\varphi \right),$$

for every r∈Ω, where

(53) $$c_{1,ex}^{0/e}=-\frac{m_{1}\alpha^{3}}{2\pi },\;c_{1,ex}^{1/e}=-\frac{m_{2}\alpha^{3}}{2\pi }\;\mathrm{and}\;c_{1,ex}^{1/o}=-\frac{m_{3}\alpha^{3}}{2\pi },$$

which leads (through (13)) to

(54) $$H_{3}^{s}\left(r\right)=c_{1,ex}^{0/e}\nabla \left[r^{-2}Y_{1}^{0/e}\left(\zeta ,\varphi \right)\right]+c_{1,ex}^{1/e}\nabla \left[r^{-2}Y_{1}^{1/e}\left(\zeta ,\varphi \right)\right]+c_{1,ex}^{1/o}\nabla \left[r^{-2}Y_{1}^{1/o}\left(\zeta ,\varphi \right)\right]$$

or

(55) $$H_{3}^{s}\left(r\right)=\frac{1}{2\pi }{\left(\frac{\alpha }{r}\right)}^{3}\left[\left(2m\cdot \hat{r}\right)\hat{r}-\left(m\cdot \hat{\zeta }\right)\hat{\zeta }-\left(m\cdot \hat{\varphi }\right)\hat{\varphi }\right],$$

where $\left(\hat{r},\hat{\zeta },\hat{\varphi }\right)$ denote the unit normal vectors [5] of the spherical geometry. This concludes the recovering of the magnetic low-frequency fields.

Our next step includes the finding of the corresponding low-frequency electric fields, beginning with $E_{1}^{s}$, so

(56) $$\begin{array}{cc} E_{1}^{s}\left(r\right) & =-\frac{1}{2}\sqrt{\frac{\mu }{\varepsilon }}\sum_{l=0}^{\infty }\sum_{m=0}^{l}\sum_{q=e,o}\left\{\nabla u_{l,ex}^{m/q}\left(r\right)\times \left[b_{l,ex}^{m/q}-\left.\frac{l\alpha^{2l+1}}{l+1}\frac{\left(l-m\right)!}{\left(l+m\right)!}\varepsilon_{m}r\left(\frac{m}{4\pi }\cdot \nabla_{r_{0}}u_{l,ex}^{m/q}\left(r_{0}\right)\right)\right]\right.\right\}\\ & =-\frac{1}{2}\sqrt{\frac{\mu }{\varepsilon }}\sum_{l=0}^{\infty }\sum_{m=0}^{l}\sum_{q=e,o}\left\{\nabla \left(r^{-\left(l+1\right)}Y_{l}^{m/q}\left(\zeta ,\varphi \right)\right)\times \left[b_{l,ex}^{m/q}-\left.\frac{l\alpha^{2l+1}}{l+1}r\left(\frac{m}{4\pi }\cdot \nabla_{r_{0}}\rho_{l,ex}^{m/q}\left(r_{0}\right)\right)\right]\right.\right\}, \end{array}$$

which is readily recovered from (37), using (14). Note that the constant coefficients $b_{l,ex}^{m/q}$ for l≥0, m=0,1,2,…,l and q=e,o are known from the previous magnetic problem for the evaluation of $H_{2}^{s}$, since it is interconnected with the current electric problem for the calculation of $E_{1}^{s}$. Our final task involves the evaluation of $E_{3}^{s}$, which is connected with the solution in (56), reading

(57) $$E_{3}^{s}\left(r\right)=X_{3}^{s}\left(r\right)-\frac{3}{2\pi }\underset{\Omega }{∭}\frac{E_{1}^{s}\left(r^{\prime }\right)}{\left|r-r^{\prime }\right|}d\Omega^{\prime }$$

for every r∈Ω, given in terms of the fundamental solution of Laplace’s equation, which is

(58) $$\begin{array}{cc} \Delta \left[-\frac{3}{2\pi }\underset{\Omega }{∭}\frac{E_{1}^{s}\left(r^{\prime }\right)}{\left|r-r^{\prime }\right|}d\Omega^{\prime }\right] & =6\underset{\Omega }{∭}\Delta \left(-\frac{1}{4\pi \left|r-r^{\prime }\right|}\right)E_{1}^{s}\left(r^{\prime }\right)d\Omega^{\prime }\\ & =6\underset{\Omega }{∭}\delta \left(r-r^{\prime }\right)E_{1}^{s}\left(r^{\prime }\right)d\Omega^{\prime }=6E_{1}^{s}\left(r\right), \end{array}$$

matching (15), where $\delta \left(r-r^{\prime }\right)$ is the well-known delta function, while

(59) $$X_{3}^{s}\left(r\right)=\sum_{l=0}^{\infty }\sum_{m=0}^{l}\sum_{q=e,o}d_{l,ex}^{m/q}r^{-\left(l+1\right)}Y_{l}^{m/q}\left(\zeta ,\varphi \right).$$

At this stage, we apply a tricky mathematical technique, according to which we write the integral within (57), using the limiting procedure

(60) $$\begin{array}{cc} -\frac{3}{2\pi }\underset{\Omega }{∭}\frac{E_{1}^{s}\left(r^{\prime }\right)}{\left|r-r^{\prime }\right|}d\Omega^{\prime }=-\frac{3}{2\pi }\{ & \underset{e\to 0}{\lim}\int_{0}^{2\pi }\int_{-1}^{1}\int_{\alpha }^{r_{0}-e}\frac{E_{1}^{s}\left(r^{\prime }\right)}{\left|r-r^{\prime }\right|}{r^{\prime }}^{2}dr^{\prime }d\zeta^{\prime }d\varphi^{\prime }\\ & +\underset{e\to 0}{\lim}\int_{0}^{2\pi }\int_{-1}^{1}\int_{r_{0}-e}^{r_{0}+e}\frac{E_{1}^{s}\left(r^{\prime }\right)}{\left|r-r^{\prime }\right|}{r^{\prime }}^{2}dr^{\prime }d\zeta^{\prime }d\varphi^{\prime }\\ & \left.+\underset{e\to 0}{\lim}\int_{0}^{2\pi }\int_{-1}^{1}\int_{r_{0}+e}^{+\infty }\frac{E_{1}^{s}\left(r^{\prime }\right)}{\left|r-r^{\prime }\right|}{r^{\prime }}^{2}dr^{\prime }d\zeta^{\prime }d\varphi^{\prime }\right\}, \end{array}$$

Hence, a careful analysis by virtue of the orthogonality relationship (25), ends up with the formula

(61) $$-\frac{3}{2\pi }\underset{\Omega }{∭}\frac{E_{1}^{s}\left(r^{\prime }\right)}{\left|r-r^{\prime }\right|}d\Omega^{\prime }=\sum_{l=0}^{+\infty }\sum_{m=0}^{l}\sum_{q=e,o}\left[s_{l,in}^{m/q}r^{l}+s_{l,ex}^{m/q}r^{-\left(l+1\right)}+S_{l}^{m/q}\left(r\right)\right]Y_{l}^{m/q}\left(\zeta ,\varphi \right),$$

where functions $s_{l,in}^{m/q}$, $s_{l,ex}^{m/q}$ and $S_{l}^{m/q}$ are determined in view of the already calculated field $E_{1}^{s}$ via

(62) $$s_{l,in}^{m/q}=-\frac{3}{2\pi }\frac{\left(l-m\right)!}{\left(l+m\right)!}\varepsilon_{m}\underset{e\to 0}{\lim}\int_{0}^{2\pi }\int_{-1}^{1}\int_{\alpha }^{r_{0}-e}{r^{\prime }}^{-\left(l+1\right)}Y_{l}^{m/q}\left(\zeta^{\prime },\varphi^{\prime }\right)E_{1}^{s}\left(r^{\prime }\right){r^{\prime }}^{2}dr^{\prime }d\zeta^{\prime }d\varphi^{\prime },$$

(63) $$s_{l,ex}^{m/q}=-\frac{3}{2\pi }\frac{\left(l-m\right)!}{\left(l+m\right)!}\varepsilon_{m}\underset{e\to 0}{\lim}\int_{0}^{2\pi }\int_{-1}^{1}\int_{r_{0}+e}^{+\infty }{r^{\prime }}^{l}Y_{l}^{m/q}\left(\zeta^{\prime },\varphi^{\prime }\right)E_{1}^{s}\left(r^{\prime }\right){r^{\prime }}^{2}dr^{\prime }d\zeta^{\prime }d\varphi^{\prime }$$

and

(64) $$S_{l}^{m/q}\left(r\right)=-\frac{3\left(2l+1\right)}{8\pi^{2}}\varepsilon_{m}\frac{\left(l-m\right)!}{\left(l+m\right)!}\int_{0}^{2\pi }\int_{-1}^{1}\left[\underset{e\to 0}{\lim}\int_{0}^{2\pi }\int_{-1}^{1}\int_{r_{0}-e}^{r_{0}+e}\frac{E_{1}^{s}\left(r^{\prime }\right)}{\left|r-r^{\prime }\right|}{r^{\prime }}^{2}dr^{\prime }d\zeta^{\prime }d\varphi^{\prime }\right]Y_{l}^{m/q}\left(\zeta ,\varphi \right)d\zeta d\varphi ,$$

respectively; therefore, by substituting (59) and (61) with (62)–(64) into the field (57), we arrive at the expression

(65) $$E_{3}^{s}\left(r\right)=\sum_{l=0}^{\infty }\sum_{m=0}^{l}\sum_{q=e,o}\left\{\left[\left(d_{l,ex}^{m/q}+s_{l,ex}^{m/q}\right)r^{-\left(l+1\right)}+s_{l,in}^{m/q}r^{l}+S_{l}^{m/q}\left(r\right)\right]Y_{l}^{m/q}\left(\zeta ,\varphi \right)\right\}.$$

Once more, herein, the constants $d_{l,ex}^{m/q}=d_{l,1}^{m/q}{\hat{x}}_{1}+d_{l,2}^{m/q}{\hat{x}}_{2}+d_{l,3}^{m/q}{\hat{x}}_{3}$ for any value of l≥0, m=0,1,2,…,l and q=e,o, satisfy the three independent expressions

(66) $$\sum_{l=0}^{+\infty }\sum_{m=0}^{l}\sum_{q=e,o}\left[\sum_{j=1}^{3}{\bar{f}}_{l,j}^{m/q,\kappa }\left(r_{s},\zeta ,\varphi \right)d_{l,j}^{m/q}-{\bar{g}}_{l}^{m/q,\kappa }\left(r_{s},\zeta ,\varphi \;;r_{0}\right)\right]=0,\;\kappa =1,2,3,$$

wherein ${\bar{f}}_{l,j}^{m/q,\kappa }\left(r_{s},\zeta ,\varphi \right)$ and ${\bar{g}}_{l}^{m/q,\kappa }\left(r_{s},\zeta ,\varphi \;;r_{0}\right)$ are complicated functions of ζ,φ in terms of $P_{l}^{m}\left(\zeta \right),\;f_{m}^{q}\left(\varphi \right)$, where, again, in order for this work to be complete and independent, we give their form here. Consequently, for κ=1, we obtain

(67) $${\bar{f}}_{l,1}^{m/q,1}\left(\zeta ,\varphi \right)=\alpha^{-\left(l+2\right)}\left[-\left(l+1\right)\zeta P_{l}^{m}\left(\zeta \right)+\left(1-\zeta^{2}\right)P_{l}^{m^{\prime }}\left(\zeta \right)\right]f_{m}^{q}\left(\varphi \right),$$

(68) $$\begin{array}{cc} {\bar{f}}_{l,2}^{m/q,1}\left(\zeta ,\varphi \right)=\alpha^{-\left(l+2\right)} & \left[\left(-\left(l+1\right)P_{l}^{m}\left(\zeta \right)-\zeta P_{l}^{m^{\prime }}\left(\zeta \right)\right)\sqrt{1-\zeta^{2}}\cos\varphi f_{m}^{q}\left(\varphi \right)\right.\\ & \left.-{\left(1-\zeta^{2}\right)}^{-1/2}P_{l}^{m}\left(\zeta \right)\sin\varphi f_{m}^{q^{\prime }}\left(\varphi \right)\right], \end{array}$$

(69) $${\bar{f}}_{l,3}^{m/q,1}\left(\zeta ,\varphi \right)=\alpha^{-\left(l+2\right)}\left[\left(-\left(l+1\right)P_{l}^{m}\left(\zeta \right)-\zeta P_{l}^{m^{\prime }}\left(\zeta \right)\right)\sqrt{1-\zeta^{2}}\sin\varphi f_{m}^{q}\left(\varphi \right)\right.\;\left.+{\left(1-\zeta^{2}\right)}^{-1/2}P_{l}^{m}\left(\zeta \right)\cos\varphi f_{m}^{q^{\prime }}\left(\varphi \right)\right],$$

and

(70) $${\bar{g}}_{l}^{m/q,1}\left(\zeta ,\varphi \;;r_{0}\right)=-\left[s_{l,in}^{m/q}+\frac{1}{\alpha^{l}}S_{l}^{m/q}\left(\alpha \right)\right]\cdot \nabla_{\alpha }\left[\alpha^{l}Y_{l}^{m/q}\left(\zeta ,\varphi \right)\right]-s_{l,ex}^{m/q}\cdot \nabla_{\alpha }\left[\frac{1}{\alpha^{l+1}}Y_{l}^{m/q}\left(\zeta ,\varphi \right)\right]-\;-\hat{r}\cdot \left[{S_{l}^{m/q}}^{\prime }\left(\alpha \right)-\frac{l}{\alpha }S_{l}^{m/q}\left(\alpha \right)\right]Y_{l}^{m/q}\left(\zeta ,\varphi \right),$$

in which $\nabla_{\alpha }$ is the gradient operator, where instead of taking the derivative over the regular r-variable, we use differentiation with respect to α. Continuing for κ=2, we have

(71) $${\bar{f}}_{l,1}^{m/q,2}\left(\zeta ,\varphi \right)=0,$$

(72) $${\bar{f}}_{l,2}^{m/q,2}\left(\zeta ,\varphi \right)=\alpha^{-\left(l+1\right)}\sin\varphi Y_{l}^{m/q}\left(\zeta ,\varphi \right),$$

(73) $${\bar{f}}_{l,3}^{m/q,2}\left(\zeta ,\varphi \right)=-\alpha^{-\left(l+1\right)}\cos\varphi Y_{l}^{m/q}\left(\zeta ,\varphi \right)$$

and

(74) $${\bar{g}}_{l}^{m/q,2}\left(\zeta ,\varphi \;;r_{0}\right)=-\hat{r}\times \left[\left(s_{l,in}^{m/q}+I{\;}_{l}^{m/q}\;\left(\alpha ,\zeta ,\varphi \;;r_{0}\right)\right)\alpha^{l}+s_{l,ex}^{m/q}\alpha^{-\left(l+1\right)}+S_{l}^{m/q}\left(\alpha \right)\right]\cdot \hat{\zeta }\;Y_{l}^{m/q}\left(\zeta ,\varphi \right),$$

while, for κ=3, we obtain

(75) $${\bar{f}}_{l,1}^{m/q,3}\left(\zeta ,\varphi \right)=-\alpha^{-\left(l+1\right)}\sqrt{1-\zeta^{2}}Y_{l}^{m/q}\left(\zeta ,\varphi \right),$$

(76) $${\bar{f}}_{l,2}^{m/q,3}\left(\zeta ,\varphi \right)=\alpha^{-\left(l+1\right)}\zeta \cos\varphi Y_{l}^{m/q}\left(\zeta ,\varphi \right),$$

(77) $${\bar{f}}_{l,3}^{m/q,3}\left(\zeta ,\varphi \right)=\alpha^{-\left(l+1\right)}\zeta \sin\varphi Y_{l}^{m/q}\left(\zeta ,\varphi \right)$$

and

(78) $${\bar{g}}_{l}^{m/q,3}\left(\zeta ,\varphi \;;r_{0}\right)=-\hat{r}\times \left[\left(s_{l,in}^{m/q}+I{\;}_{l}^{m/q}\;\left(\alpha ,\zeta ,\varphi \;;r_{0}\right)\right)\alpha^{l}+s_{l,ex}^{m/q}\alpha^{-\left(l+1\right)}+S_{l}^{m/q}\left(\alpha \right)\right]\cdot \hat{\varphi }\;Y_{l}^{m/q}\left(\zeta ,\varphi \right),$$

which are provided for any value of $\zeta \in \left[-1,1\right]$ and $\varphi \in \left[0,2\pi \right)$. As already mentioned, the functions $s_{l,in}^{m/q}$, $s_{l,ex}^{m/q}$ and $S_{l}^{m/q}$ yield the expressions (62)–(64), while the convenient expression

(79) $$I{\;}_{l}^{m/q}\;\left(\alpha ,\zeta ,\varphi \;;r_{0}\right)=-\frac{3}{4\pi }\sqrt{\frac{\mu }{\varepsilon }}m\times \left[r\left(r,\zeta ,\varphi \right)-r_{0}\right]\rho_{l,ex}^{m/q}\left(r_{0}\right)$$

for l≥0, m=0,1,2,…,l and q=e,o refers to the dipole’s position and, as usual, all derivatives denoted by the prime are with respect to the argument. Thereafter, we proceed with the same manner as previously and we expand the functions (67)–(78) in terms of surface spherical harmonics. Since they belong to the subspace that they produce, we substitute the result into the relations (66) and the outcome is handled with the aid of orthogonality (25), so as to recover the components $d_{l,j}^{m/q}$ (j=1,2,3) of the constant coefficient $d_{l,ex}^{m/q}$ for l≥0, m=0,1,2,…,l and q=e,o. At this stage, we are readily obliged to mention that the gradient differential operator in spherical geometry assumes the form that is given by

(80) $$\nabla =\sum_{j=1}^{3}{\hat{x}}_{j}\frac{\partial }{\partial x_{j}}=\hat{r}\frac{\partial }{\partial r}-\frac{\sqrt{1-\zeta^{2}}}{r}\hat{\zeta }\frac{\partial }{\partial \zeta }+\frac{1}{r\sqrt{1-\zeta^{2}}}\hat{\varphi }\frac{\partial }{\partial \varphi },$$

which, in our project, is utilized over the position of the magnetic dipole source $r_{0}$ as $\nabla_{r_{0}}$, concluding, in this way, the theoretical development of the presented physical and mathematical situation.

Recapitulating our analysis, we have retrieved, in a closed-type analytical fashion, the surviving (for n=0,1,2,3) low-frequency terms of the scattered magnetic $H_{0}^{s}$, $H_{2}^{s}$ and $H_{3}^{s}$ (see (35), (37) and (55), respectively) and electric $E_{1}^{s}$ and $E_{3}^{s}$ (see (56) and (65), respectively) fields, as they are under consideration in the spherical domain Ω from (28), considering well-posed boundary value problems, while $H_{1}^{s}=E_{0}^{s}=E_{2}^{s}=0$, bearing in mind that terms of a higher order (for n≥4) are considered of minor significance since they do not contribute to the field calculation. In order to separate the real from the imaginary part in the scattered fields, we substitute the wave number $k=\omega \sqrt{\varepsilon \mu }$ of the embedding perfect dielectric medium into (7) and (8), keeping the field orders of interest, in order to arrive at the scattered electromagnetic fields

(81) $$H^{s}=\left[H_{0}^{s}-\frac{{\left(\omega \sqrt{\varepsilon \mu }\right)}^{2}}{2}H_{2}^{s}\right]+\left[-\frac{{\left(\omega \sqrt{\varepsilon \mu }\right)}^{3}}{6}H_{3}^{s}\right]i+○\left({\left(ik\right)}^{4}\right)\;\mathrm{for}\;r\in \Omega$$

and

(82) $$E^{s}=\omega \sqrt{\varepsilon \mu }\left[E_{1}^{s}-\frac{{\left(\omega \sqrt{\varepsilon \mu }\right)}^{2}}{6}E_{3}^{s}\right]i+○\left({\left(ik\right)}^{4}\right)\;\mathrm{for}\;r\in \Omega .$$

Obviously, as it is revealed from (81) and (82), the scattered magnetic field is complex-valued, while the scattered electric field is purely imaginary, which is dictated by the relevant low-frequency terms.

## 4. Numerical Implementation

**Figure 2:**
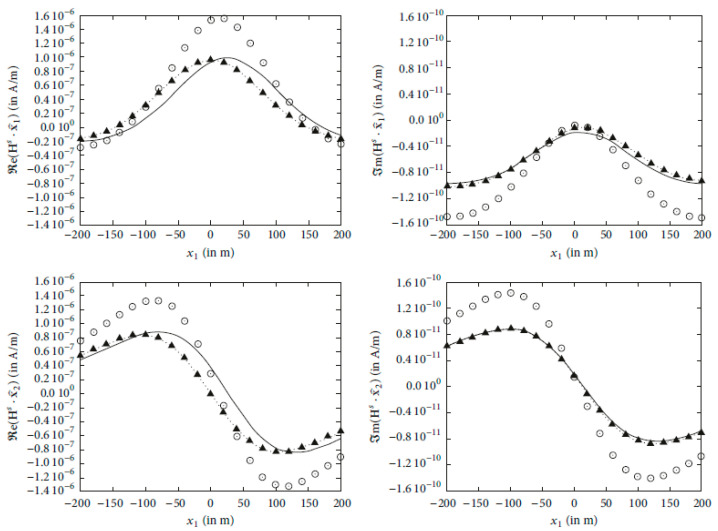
The real (**left** column) and imaginary (**right** column) parts of the components of the low-frequency (ω=50 Hz) scattered magnetic field $H^{s}$ in A/m, which is excited by a vertically orientated ($m_{2}=m_{3}=0$) magnetic dipole source $m=m_{1}{\hat{x}}_{1}$ of strength $m_{1}=4\pi \times 10^{3}\;A\;m^{2}$ and location at $r_{0}=\left(200\;m,0,0\right)$. The measurement is taken on a line at $\left(\left[-200,200\right]m,200\;m,0\right)$, obtaining the field in m on a vertical axis parallel to $x_{1}$-axis for different expansion degrees, keeping the series terms for L=0 (⊙), L=1 (···▲···) and L=2 (**––**). The $x_{3}$-components are all vanishing and are represented by a straight line passing from zero.

In order to demonstrate the efficiency of the above analysis by means of assessing the validity and the accuracy of the produced formulae, we intend to provide plots that display the numerical behavior of the scattered magnetic field (75), i.e., the field $H^{s}$ in A/m, which is usually measured. We approximate the low-frequency expansions up to the third degree (n=0,1,2,3) and we utilize the associated magnetic counterparts (30), (32) and (50), posing an upper limit L to the infinite series (l=0,1,…,L), which is appropriately chosen until convergence is obtained. We adopt the spherical geometry in Figure 1 by considering a perfectly conducting sphere of extremely large conductivity and radius α=50 m. The spherical body is embedded in a homogeneous vacuum environment (air) of dielectric permittivity $\varepsilon ≃\varepsilon_{0}=8.854\times 10^{-12}\;F/m$ and magnetic permeability $\mu ≃\mu_{0}=4\pi \times 10^{-7}\;N/A^{2}$, whilst the electric conductivity is then assumed to be approximately zero. Thereafter, we illuminate the object with a vertically orientated dipole source $m=m_{1}{\hat{x}}_{1}$ of strength $m_{1}=4\pi \times 10^{3}\;A\;m^{2}$, which is set at $r_{0}=\left(200\;m,0,0\right)$ and radiates at the low frequency of ω=50 Hz. Bearing in mind this discussion, the scattered magnetic field $H^{s}$ is evaluated along a fictitious measuring line, being positioned at $\left(\left[-200,200\right]m,200\;m,0\right)$, and in Figure 2, we provide graphical illustrations for the real (left column) and imaginary (right column) parts of the approximated scattered magnetic field under consideration in A/m. During the numerical implementation and in order to show the influence of the series terms that we keep, we present plots for L=0, L=1 and L=2, observing that the $x_{3}$-components are all vanishing.

The low-frequency scattered magnetic field, which is sketched in Figure 2, is verified to attain similar behavior with the respective work in the spherical realm [7], wherein the only difference is that the surrounding is conductive, changing the concept but keeping the idea. This fact secures the credibility of the obtained results on a numerical level. On the other hand, the convergence of the infinite series expansions of the scattered magnetic low-frequency terms is readily achieved immediately, since the different diagrams in Figure 2 tend to be almost matching for l=0,1,2, corresponding to an upper limit value of L=2, which shows the reliability of the presented methodology in a quantitative level, being fast enough for possible inversion utility. Otherwise, the source location and the location of the measurement line (parallel to the $x_{1}$-axis) justify the set of the plotted graphs, which appear symmetric, as expected, due to the dipole’s position, on the direction of the $x_{1}$-axis. Finally, in an analytical level, the closed-form solutions in this project are given in a fashion that matches the procedure that is followed in many related published articles (e.g., see [14,15,16,17,18] for more details) in the literature; hence, this fact ensures the main technical steps used in this project.

## 5. Conclusions

In this work, we investigated the low-frequency approximation of the fields that are scattered by a perfectly conductive sphere, embedded in a lossless medium and excited by a far-field and arbitrarily orientated time-harmonic magnetic dipole which produces the primary electric and magnetic fields. The developed analytical methodology was based on the introduction of power series expansions of the electromagnetic fields in terms of the wave number of the medium, keeping the first four terms that are sufficient in the low-frequency spectrum, while the terms of higher orders are negligible. The classical Maxwell-type problem was transformed to a sequence of interconnected elliptic-type relationships, which are accompanied by the impenetrable boundary conditions on the surface of the scatterer, while the limiting behavior at an infinite distance was readily secured. Upon the introduction of a suitable spherical geometry, the obtained boundary value problems were readily solved in a complete analytical fashion, providing three-dimensional compact formulae, in view of infinite series expansions of spherical harmonic eigenfunctions, while the outcomes of this research were validated via a consistent numerical illustration of the measurable scattered magnetic field. The graph representation showed accordance with the literature and the behavior of the components of the field was the expected one.

In such analytical or semi-analytical approaches, we are confronted with a near-field problem, where planar skin depths are significantly larger than source–body or body–sensor distances and, therein, only diffusion phenomena occur, since conduction currents are predominant. Therefore, the solution that was given here appears to be a good approximation at low frequencies and suitably describes the behavior of a complete isotropic spherical-shaped metallic object. The advantages of the current formulation, compared to pure numerical methods, lie in the analytical expressions that yielded closed-type compact forms, involving simple analytically known constant coefficients for any order of the spherical harmonics introduced, which were into the functions. Consequently, any numerical evaluations of the fields could be very fast and can be achieved until the required convergence is obtained. This can be very useful for future inverse schemes in the low-frequency spectrum, since the localization and identification of an unknown scatterer can be effectively achieved as long as the corresponding fields are distinctly described.

## Data Availability

The original contributions presented in the study are included in the article, further inquiries can be directed to the corresponding author.

## Appendix A

The current research is developed according to the theory of low frequencies, where the nature of the illuminating source (here, the magnetic dipole m in (2), positioned at $r_{0}$ with an arbitrary orientation) determines the response of the scattered (and, obviously, the total) fields in the low-frequency regime. Therefore, here we provide the necessary mathematical material of the low-frequency behavior of the excitation incident fields that starts the procedure and controls the electromagnetic scattering problem under consideration.

The exact solution of a wave from the magnetic dipole (2) without the presence of a scatterer comes from the Green function of the non-homogeneous Maxwell’s equations with singularity at $r_{0}$ (see [4] for details); thus, the primary (incident) magnetic field assumes the form

(A1) $$H^{p}\left(r\right)=\frac{1}{4\pi }\left[\left(k^{2}+\frac{ik}{R}-\frac{1}{R^{2}}\right)m-\left(k^{2}+\frac{3ik}{R}-\frac{3}{R^{2}}\right)\frac{R⊗R\cdot m}{R^{2}}\right]\frac{e^{ikR}}{R}\;\mathrm{for}\;r\in \Omega ,$$

while the primary (incident) electric field yields

(A2) $$E^{p}\left(r\right)=\left[\frac{\omega \mu k}{4\pi }\left(1+\frac{i}{kR}\right)\frac{m\times R}{R}\right]\frac{e^{ikR}}{R}\;\mathrm{for}\;r\in \Omega ,$$

wherein $R=r-r_{0}$ and $R=\left|r-r_{0}\right|$, while the symbol “⊗” denotes the classical dyadic product. Since the operating frequency ω of the primary source is considered very low, then the wave number in the lossless environment $k=\omega \sqrt{\varepsilon \mu }$, which is proportional to ω, is very low as well, since the dielectric permittivity ε and the magnetic permeability μ of the medium are standard constants. Under this aim, we handle the wave fields (A1) and (A2) in such a way so as to obtain formulae with respect to integral powers of k. In view of this aspect, we readily apply the Maclaurin’s series expansion of the exponent $e^{ikR}$, we eliminate ω via relationship $\omega =k/\sqrt{\varepsilon \mu }$ and we perform some cumbersome, but straightforward, analytical manipulations, concluding to the corresponding low-frequency relations of the incident electromagnetic fields, i.e.,

(A3) $$H^{p}\left(r\right)=\left[H_{0}^{p}\left(r\right)+\frac{H_{2}^{p}\left(r\right)}{2}{\left(ik\right)}^{2}+\frac{H_{3}^{p}\left(r\right)}{6}{\left(ik\right)}^{3}\right]+○\left({\left(ik\right)}^{4}\right)\;\mathrm{for}\;r\in \Omega$$

and

(A4) $$E^{p}\left(r\right)=\left[E_{1}^{p}\left(r\right)\left(ik\right)+\frac{E_{3}^{p}\left(r\right)}{6}{\left(ik\right)}^{3}\right]+○\left({\left(ik\right)}^{4}\right)\;\mathrm{for}\;r\in \Omega ,$$

in which

(A5) $$H_{0}^{p}\left(r\right)=\frac{m}{4\pi }\cdot \left(\nabla ⊗\nabla \frac{1}{R}\right)\;\mathrm{for}\;r\in \Omega ,$$

(A6) $$H_{2}^{p}\left(r\right)=\frac{m}{4\pi }\cdot \left(\nabla \frac{1}{R}⊗R-\frac{\tilde{I}}{R}\right)\;\mathrm{for}\;r\in \Omega ,$$

(A7) $$H_{3}^{p}\left(r\right)=\frac{m}{4\pi }\cdot \left(-4\tilde{I}\right)\;\mathrm{for}\;r\in \Omega$$

and

(A8) $$E_{1}^{p}\left(r\right)=-\frac{m}{4\pi }\sqrt{\frac{\mu }{\varepsilon }}\times \nabla \frac{1}{R}\;\mathrm{for}\;r\in \Omega ,$$

(A9) $$E_{3}^{p}\left(r\right)=-\frac{m}{4\pi }\sqrt{\frac{\mu }{\varepsilon }}\times \frac{3R}{R}\;\mathrm{for}\;r\in \Omega ,$$

respectively, wherein $H_{1}^{p}=E_{0}^{p}=E_{2}^{p}=0$. In the sought relationships (A5)–(A9), $\tilde{I}$ denotes the unit dyadic, while we have used the fact that $\nabla \left(1/R\right)=-R/R^{3}$ and $\nabla ⊗r=\tilde{I}$. On the other hand, due to the implied low-frequency analysis, we have calculated the first four (n=0,1,2,3) important and, obviously, adequate terms for the magnetic (A3) and the electric (A4) fields, while terms of higher orders (n≥4) are neglected since k is very small. Therein, the low-frequency approximation is well justified and it is reflected to the corresponding surviving scattered fields.
